# Supplementary material for: Self-Boundary Dissolution in Meditation: A Phenomenological Investigation
Source: Brain Sci. 2021 Jun 21;11(6):819. doi: 10.3390/brainsci11060819 (PMC8235013; doi:10.3390/brainsci11060819)
Supplement: Supplementary file 1 [file brainsci-11-00819-s001.zip › brainsci-1203084-supplementary.pdf]

Supplementary Materials

1. Supplementary Methods

1.1. Self-boundary Meditation Training Program

The training program for the neurophenomenological project was designed and taught by Stephen Fulder, the founder of the Israeli society for insight meditation ("Tovana") and expert meditator in an earlier study [24,26]. It was intended to give participants a number of meditative and contemplative ways to explore sense of self and boundary and dissolution of sense of self and boundary. The training program lasted over 3 weeks and consisted of an initial full day workshop with guided meditation exercises and practical and theoretical discussion of the practice, and 3-weeks of daily home practice which included two group sessions for questions, guidance and support. Additionally, participants received recorded instructions for home practice. Presentations and meditation instructions from the workshop were recorded for documentation and were provided to those participants that could not attend the meetings. Two recordings of guided meditation sessions are available online on the project page of the Open Science Framework (https://osf.io/bsxua/).

The full day workshop included teachings and guided meditation practices intended to familiarize participants with different dimensions of the self, challenge its solidity and explore more directly the sense of boundary and its dissolution. A range of meditative techniques and scenarios were used to give participants various ways into such subtle experiences that fitted their tendencies and previous practice. The teachings were offered as ways to inner transformation as well as preparation for the experiment which would examine meditative experiences of boundary and boundarylessness. The workshop consisted of:

- An introduction to the SB research project, including an overview of the experimental setup.
- Discourse on the self in the context of Buddhist spiritual practice.
- Guided meditations focused on self/no-self (Atta/Anatta), emphasizing the experience of "selfing" as dynamic and transparent. For example exploring the experience of witnessing, thinking, knowing, owning and subject/object.
- Guided meditation focused on boundaries, such as body sensations, points of contact, sense of center and inner/outer.
- Guided meditations focused on boundarylessness, such as spaciousness, deep relaxation, relinquishing control, diffused attention, soundscape, or limitlessness.
- Guided meditations exploring the experience of limit, contracted and expanded awareness, including mindful walking exercise.
- Lying down meditation on "Big mind" and aliveness
- Several open discussions and questions and answers on meditation methods and strategies

The following group sessions were similarly focused on refining and clarifying meditative techniques allowing participants to personally choose and suggest accessible methods according to their own preference and previous experience. The training aimed to recruit each participants' own abilities while also preparing for the unfamiliar and possibly demanding conditions of practicing meditation in a lab setting, which required both rapid transitions between meditative states while in an unusual horizontal meditative posture suitable for magnetoencephalography (MEG) measurement.

The suggested daily home practice involved 45 minutes of both meditative conditions (SB+\SB-) with the help of recorded instructions where needed.

1.2. Phenomenological Glossary

This glossary was created based on an intermediate stage of analysis, in order to clarify the meaning of each category and its specific values\subcategories. Rather than a precise definition it was meant to be indicative and helpful for the raters during the characterization process. It is phrased addressing them. Each category was given a general description, a list of subcategories and an example for each.

Meditation technique

This category captures the kind of meditation being executed according to its technique. The sub-categories delineate either conscious gestures that are undertaken or the prominent realm of experience through which boundaries are experienced and altered.

SB+<br><br>Meditation technique	**Options**<br>A. Sensations scan<br>B. Feeling\visualising form of body image (defining a spatial frame)<br>C. Dwelling within body boundaries (Described with weight sensations, density, whole areas)	**SB+ Examples**<br>A. "Something within me is searching the sensations there and finds them." Dynamic, focused, sensations<br>B. "Attention is simply listening to the body and it just constructs a certain space" – define form (visual or sensual)<br>C. "There's a sense of just staying inside this space, inside the body."
SB-<br><br>Meditation technique	**Options**<br>D. Sensations (change of perspective, deformation)<br>E. Turning attention outwards<br>F. Imagination or memory of a spatial scene<br>G. Relaxation, letting go, passivity<br>H. Other: Emotion based (Metta), analytic thoughts	**SB- Examples**<br>D. "I feel this little sensations changing all the time. I try to focus on the subtle sensations – that is what melted the body boundaries."<br>E. "It's like searching for something outside through the sense of hearing, and then I turn attention."<br>F. "I had this image in my mind, of some space, that I see earth from outside, and with my inhaling there are waves coming out of there."<br>G. "It's an immediate opening, a release. There's no other mental movement of wanting or not wanting, some kind of presence of simply being."

Degree of dissolution

This category is an evaluation based on various variables that define the quality of the sense of boundaries and their dissolution. It has to do with the relationship one has with the surrounding or the clarity over the distinction between the inside and the outside? Is one's sense of self distinct or intertwined with the surrounding in some way? To what extent are the boundaries closed or permeable? How stable and prolonged was the dissolution described?

The values are here presented along with some general non-specific descriptions which are not meant to be binding but assist in providing some intuitive grasp of the scale.

Note the guidelines for identifying authentic descriptions and take into account the use of first-person pronouns and para-verbal information.

SB-<br><br>Degree of dissolution	**Scale 1-9** (for SB- only)<br>1: Boundaries are defined, closed and they distinguish oneself from the outside clearly.<br>3: Sense of relation to the surrounding, which is now included within perceptual experience. It is still clearly located and differentiated but there's a growing sense of interchange.<br>5: Sense of change in quality and location of self-boundaries: Expansion or ambiguity regarding location. Boundaries appear more permeable or indistinct as they are connected to the outside. A sense of self is still present in some distinguished way.<br>7: Greater sense of dissolution of boundaries. Sense of self is experienced as part of a greater whole, or minimized into a thinner experience of selfhood. Still maintaining a sense of space organized around a (bodily) center.<br>9: Radical dissolution of boundaries into a unified sense of spaciousness, indistinct, non-self, non-dual. Boundaries are no longer apparent in conscious experiences. Experience is generally increasingly formless.

Sense of agency - The sense that I am causing or generating an action. Sense of agency relates to the feeling of controlling attention or directing experience in some way. It has to do with a sense of being consciously active rather than passive.

SB+<br><br>SB-<br><br>Agency	**Options** for both<br>1. Active (Continuously)<br>2. Responsive (Passive with task maintenance)<br>3. Passive (non-doing)	**Examples**<br>1. "I'm really doing something on the body, trying to feel something. An action of scanning the body."<br>2. "There's much less preference to anything. All sorts of appearances of like a sudden thought or a sensation, but it doesn't stick. It doesn't home in on anything." Maintaining concentration.<br>3. "I don't have any tasks right now, there's nothing I need to maintain… Yeah it's mostly freedom of a task, I feel." Surrendering, relaxation

Affective valence - Emotional state, valenced feelings, affective or existential stance towards being within boundaries or dissolving them.

SB+<br><br>SB-<br><br>Affective valence	**Options**<br><br>Positive (Stable, safe, familiar, pleasant, blissful)<br>Neutral<br>Mixed (Various opposing emotions)<br>Slightly Negative (Stress, uneasy, unpleasant)<br>Highly negative (intimidating, much tension, unsafe)

Attentional mode - Quality of attention, how it is used throughout the meditation. Still or dynamic, focused or wide. Sometimes described directly although often appears implicitly in relation to described objects of attention.

SB+<br><br>Attention	**Options**<br>1. Focused, Dynamic<br>2. Wide, Dynamic (Attention turned to 'beyond' \ Changing imagined content)<br>3. Wide, static<br>4. Formless	**SB+ Examples**<br>1. "There's a sensation on the skin that surrounds it, like different areas according to my wandering across the length of the body, of more attention, if it's in the feet, the back, the hands, neck, head. Like people do in the end of a yoga class, scanning the body."<br>2. "I felt the boundary but not from the outside and not in its outline, but… I felt the thigh as a thigh, or say, I felt my whole body as my whole body – the entire body… Yeah some kind of solidity."

SB-<br><br>Attention		**SB- Examples**<br>1. "I feel the little sensations changing all the time in different areas around my body." Specific objects of perception<br>2. "I open attention to the room around me, and I can really sense what surrounds me, the bed, the sounds."<br>3. "Attention opens, it's wide, much more round, and it's not engaged in making distinctions."<br>4. "A moment of disorientation, I don't know what's happening, and then there's something else." "A ray of light, being part of space".

Location -The spatial structuring of awareness through space and in relation to one's own location within it. Sense of self-location ('where I am') and spatial frame of reference of surrounding ('what else is present out there').
General sense of space changes drastically between subjects and states. How much of the outside is present in conscious experience, or how focused is it only on bodily space. How much does it differ from the predominant framing of experience as centered in the body (or the head), and surrounded by the external environment. How limited and structured is it, as opposed to vast and formless. Options here are defined in general terms to allow grouping of different yet somewhat similar experiences.

SB+<br><br>Location	**Options**<br>1. Self-location - within body, Spatial frame of reference: Body bounded, only immediate surrounding (clothes, bed)<br>2. Body & wider surrounding (room)<br>3. Expansion into vast space - significant change<br>4. Abstract self-world - radical change<br>5. Other, Ambiguous space	**SB+ Examples**<br>1. "I feel the observer is more located in the area of my face, or my chest… my sense of space is irrelevant – only the body and its presence. My posture lying down.", "A feeling of inward contraction - I'm inside my body."<br>2. "I was interested in space in which the experience of the body arises, and that's… and then it's like it was more holistic. Maybe a little… both internal and external. The experience of the body arises within this."
SB-<br><br>Location		**SB- Examples**<br>1. "The focus points toward superficial body sensations. I know where I am, and then I see the boundary is not rigid, there's movement there, indistinctness"<br>2. "My attention shifted toward being more in space which is also the body but it's also around the body. It's above and below."<br>3. "There are waves coming out of me outwards. Something expands into space and merges with it. Part of the time I was in some image of something much more vast."<br>4. "It's some kind of endless space in which there's not much meaning to form. There's no meaning to shapes when you observe things from a distance. But it's not like you're non-existent but you're part of something, a flow, an energy, light, wave. [3 seconds pause] But there's no meaning to your form, to light. There's no form."

1st person perspective - The ongoing pre-reflective sense of being a subjective agent distinct from perceived objects, as opposed to a non-dual state in which no such distinction is experienced. Usually relevant only in deep meditation. Sometimes described as a feeling of presence within space, rather than perceived space.

SB-<br><br><u>1st person perspective</u>	**<u>Options</u>**<br>1. Normal<br>2. Intermediate<br>3. Non-dual state	**<u>SB- Examples</u>**<br>2. Unstable sense of observer in relation to perceptual objects or space<br>3. "There's no attention, no experience. Or I don't know what there is. I know at some stage attention comes back to some experience but at some point there's none.

<u>Body sensations</u> - The category of body sensations reflects the salience of sensations within perceptual experience. Rather than capturing any specific quality of sensation, it generally distinguishes participants according to the clarity and distinctness with which sensations were or were not experienced. Notice the specificity of language used, or specific body parts or certain sensations, as opposed to a general sense of encompassing feelings.

SB+<br><br>SB-<br><br><u>Body Sensations</u>	**<u>Options</u>**<br>1. Prominent<br>2. Indistinct bodily sensations<br>3. Dissociated (flow, floating, non-bodily)

2. Supplementary Table

Table S1. Fuzzy Kappa for rater agreement.

Condition	Category	Observed Agreement	Expected Agreement	Fuzzy Kappa	Lower CI	Upper CI
SB-	Agency	0.761	0.333	0.641	0.464	0.811
	Location	0.772	0.294	0.676	0.518	0.817
	FPP	0.761	0.433	0.578	0.343	0.786
	Attention	0.717	0.344	0.569	0.374	0.743
	Affect	0.855	0.462	0.731	0.544	0.908
	Body	0.685	0.378	0.493	0.272	0.693
	Technique	0.779	0.355	0.657	0.520	0.793
SB+	Agency	0.870	0.630	0.648	0.429	0.865
	Location	0.902	0.781	0.554	0.239	0.846
	Attention	0.750	0.404	0.581	0.380	0.774
	Affect	0.859	0.341	0.786	0.627	0.935
	Body	0.913	0.802	0.560	0.087	0.954
	Technique	0.779	0.476	0.578	0.404	0.746

Fuzzy Kappa with 95% confidence intervals (CI) as well as observed and expected agreement for each condition and category. Calculations are based on Kirilenko & Stepchenkova (2016).

3. Supplementary Figures

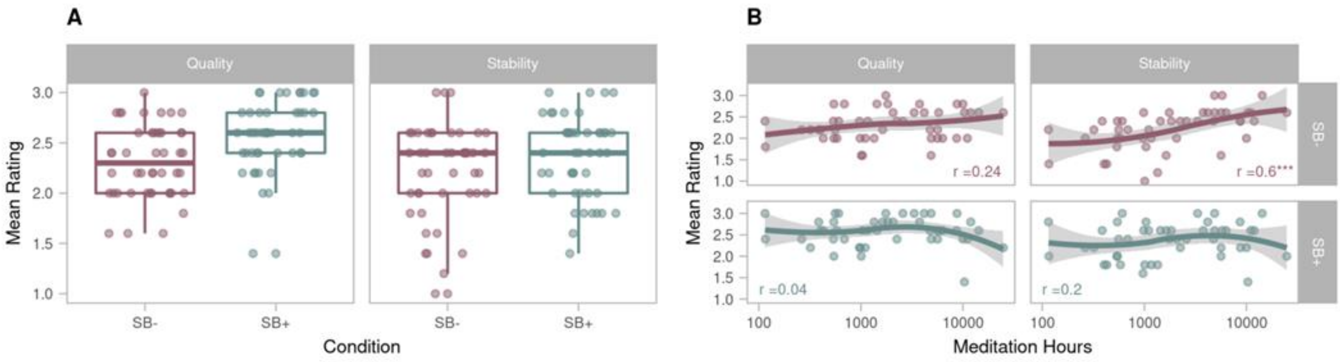

Figure S1. Self-rated depth and stability. (Panel A: Boxplots of depth and stability rated on a 1-3 scale. The bold line shows the median, boxes indicate first and third quartiles (the 25th and 75th percentiles) and whiskers show 1.5 * inter-quartile range from the hinge. Panel B: Correlation coefficient and fitted line for the relationships between lifetime hours of meditation and mean ratings. Statistics for the correlations are adjusted for multiple tests usign Bonferroni correction.).

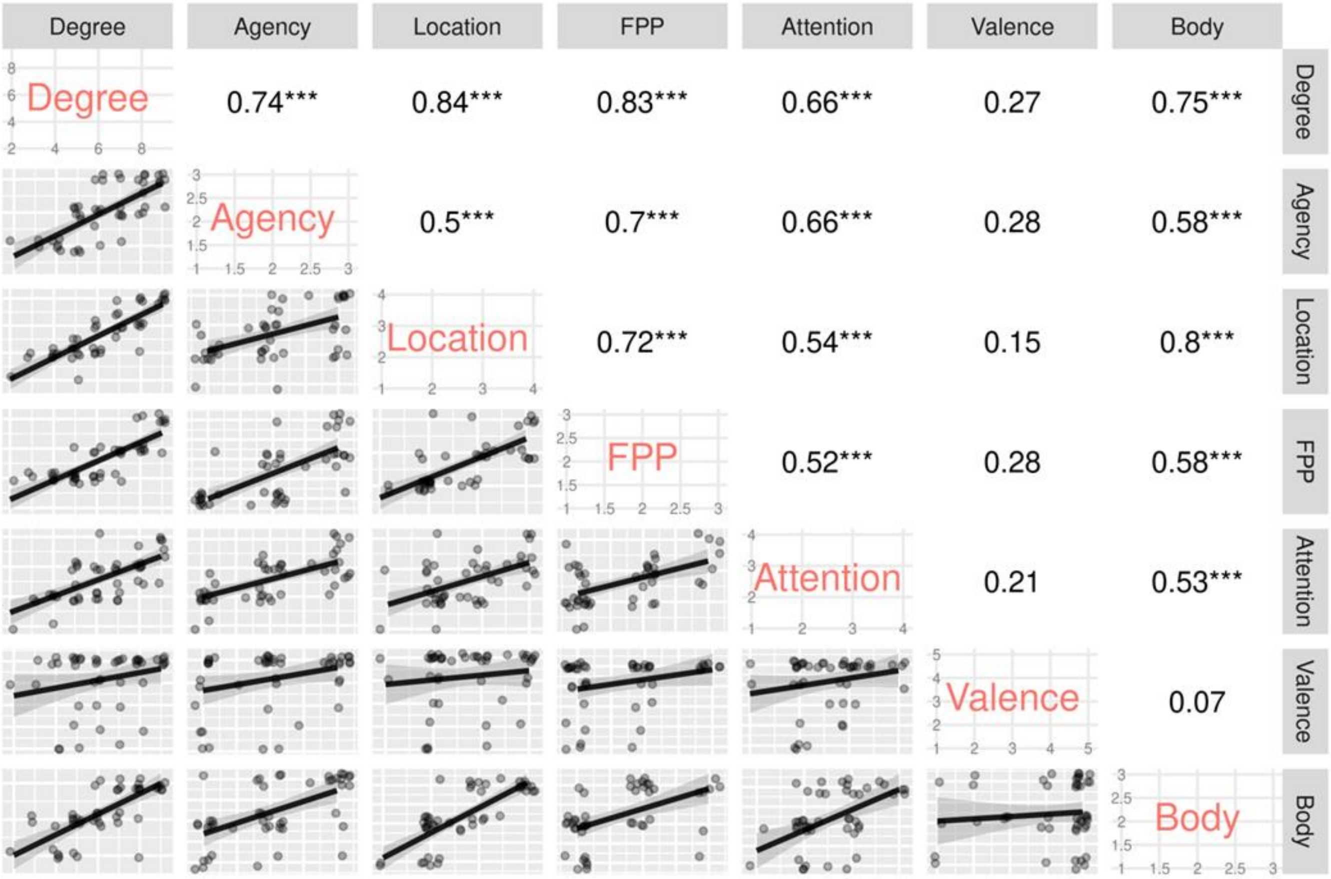

Figure S2. Relationships between the phenomenological dimensions in SB−. Notes: A random jitter was applied to make overlapping values better visible. Degree refers to degree of dissolution (DD_r). As these correlations served exploratory and descriptive purpose, no Bonferroni correction was applied.

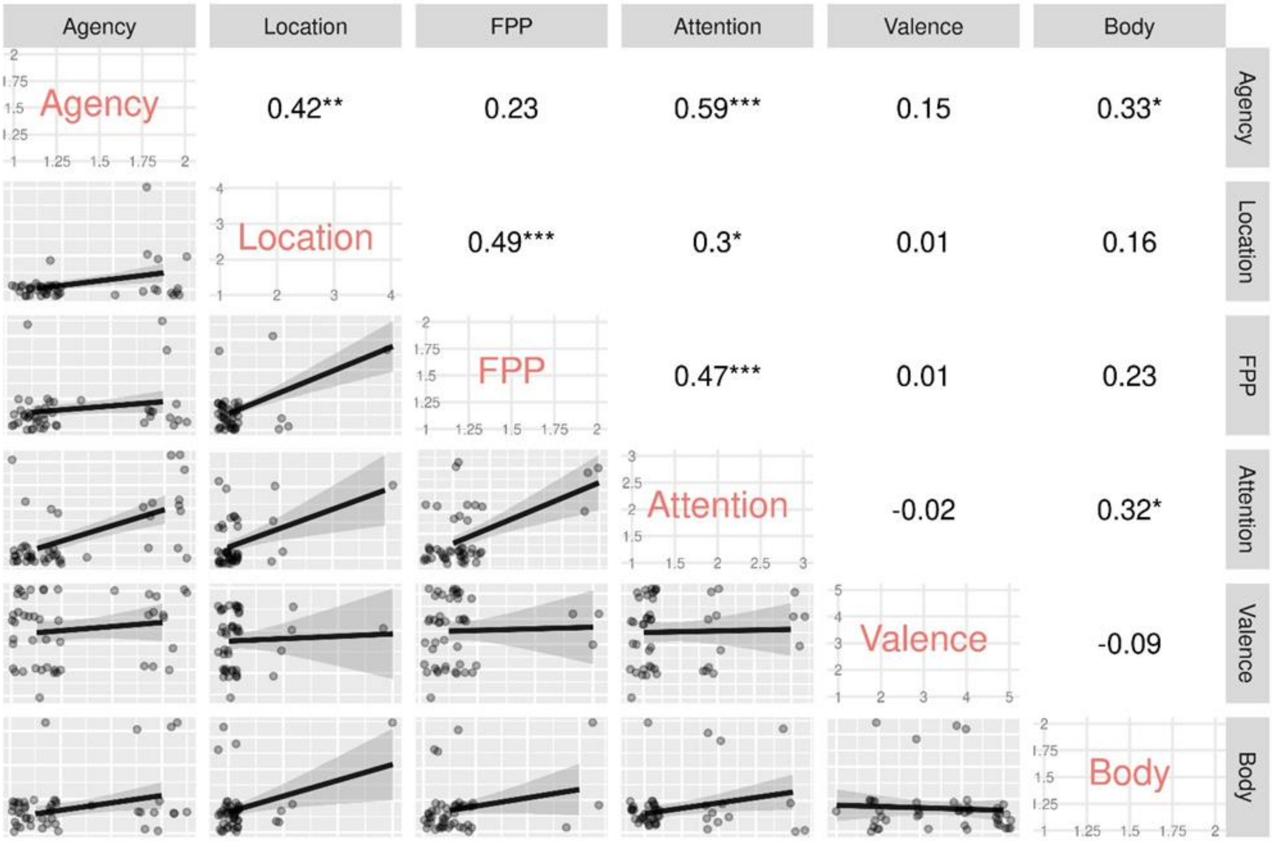

Figure S3. Relationships between the phenomenological dimensions in SB+. Notes: A random jitter was applied to make overlapping values better visible. As these correlations served exploratory and descriptive purpose, no Bonferroni correction was applied. Note that due to the low variance of the SB+ categories, the reported correlation coeifficients and significance levels are likely to be unreliable.
